# On Typhoid Fever

**Published:** 1906-06-02

**Authors:** 


### ON TYPHOID FEVER.

This article upon some aspects of typhoid fever is not intended to be an exhaustive description of the disease, but merely to draw attention to certain of its phases upon which recent experience has thrown fresh light, and to which, perhaps, in a more complete survey, but little notice would be attracted. The definite points which are dealt with are as follows:—

The causes which modify the invasion of typhoid fever producing a sudden rather than a gradual onset.

The meaning of convulsions in the course of the disease and their comparatively slight prognostic importance.

Delayed Widal reaction and an alternative method of diagnosis based on the early appearance of typhoid bacilli in the blood.

Some points in the early recognition of perforation and its effect upon the blood-pressure, with special reference to the possibility of surgical treatment.

**Sudden Onset.**—The onset of typhoid fever is commonly regarded as being one of the most constant features in a disease which in other respects presents infinite variations; but the gradual invasion, the steadily rising temperature and the increasing malaise which are usually indicative of the early stages of typhoid fever may be replaced by an onset almost dramatic in its suddenness. Several conditions may combine to alter the constancy of the initial phase, the pre-existence or coincidence of certain other maladies may have a marked influence in producing a sudden onset. Malaria, in particular, may so modify the attack that the symptoms are ushered in by a *rigor*, which in the malarial subject may scarcely command attention or lead the patient to seek advice, except in the case of one who has been free from symptoms of malaria, when it may suggest some serious return of the old complaint: for the victim of malaria, although no longer liable to the periodic attacks of ague, retains for many years this idiosyncrasy that any fresh malady accompanied by pyrexia, whether trifling or serious, is apt to develop with a rigor and the appearances of the former disease. The coincidence of one of the other epidemic fevers, influenza, scarlet fever, or diphtheria, may alter the initial stages to such an extent as at first to baffle diagnosis. In a recognised outbreak of typhoid fever this need not prove misleading; but in a sporadic or imported case, the diagnosis will often only be suggested by the persistence of the fever or the subsequent development of other signs; and of these none is more reliable than the appearance of the "rose spots" which are rarely, if ever, absent from a case of true typhoid throughout its whole course, and when seen may be regarded as conclusive even to the point of contradicting a negative Widal reaction.

**Convulsions in Typhoid Fever.**—The appearance of this symptom, although rare, is calculated to give rise to alarming apprehensions, and its meaning is often obscure; but the import of convulsions is not, as a rule, very grave, in the majority of the few cases in which they have occurred they have been manifestations of the toxæmia of the disease and have not led on to a fatal termination; they rarely point to cerebral complications, in fact, in Osler's experience, meningitis accompanying typhoid fever has not generally been attended by this symptom. The cause of convulsions has, it is true, been found to be thrombosis of the branches of the middle cerebral artery, which ended fatally, but this event is so rare as to be scarcely looked for; occasionally they may betoken a cystitis, but such a condition is by no means so dangerous when caused by the typhoid infection as under other circumstances, and will as a rule yield readily to treatment with urotropine. In general, a single convulsion, occurring without other grave symptoms, need not be regarded as an indication of specially ill omen. The prognosis of the particular attack remains, speaking broadly, unaltered, and the gravity of the case may be judged from its other manifestations.

**Delayed Widal Reaction.**—Experience is beginning to show that it is by no means uncommon for the Widal reaction not to prove positive in a case of typhoid fever until late in the course of the disease; it may happen that a new clinical distinction between the typhoid and paratyphoid infections will be invoked to explain some of the unsatisfactory negative reports which the serum reaction from time to time provides; at present the difference is in the province of bacteriology rather than of clinical medicine, the treatment remains identical even after the diagnosis of paratyphoid fever has been made, and the importance of preventing the spread of infection is no less imperative. For the present the clinician is content to recognise more latitude of variation in the symptoms of typhoid fever than the bacteriologist can admit in the characteristics of typhoid bacilli.

**Bacilli in the Blood.**—A practical point, however, in the diagnosis of those obscure cases in which the Widal reaction fails to give a conclusive result has recently been demonstrated which may prove of the utmost value, namely, that bacilli are almost always present in the blood before the serum reaction occurs. In the majority of cases the bacteriological examination of the blood for bacilli is unnecessary and difficult of performance, but there is a striking agreement in the statements of various observers as to the early appearance of bacilli in the blood, so that it appears that this "blood-culture" method of diagnosis is applicable so soon as the patient exhibits a febrile temperature. The technique of the method is difficult, and can only be performed by those who have some knowledge of bacteriology, for its successful performance depends to a great extent upon preventing the coagulation of the blood when it is withdrawn; this is accomplished by admixture with a small quantity of ox-bile and subsequent culture in a peptone-glycerine-bile medium with sub-cultivation on agar plates, as described by Dr. Conradi.[1] Still, in an important case where the diagnosis remains obscure after the Widal reaction has been found inconclusive or where an early and definite opinion as to the nature of the disease is urgent, this method (which may be carried out in from 26 to 32 hours) should be borne in mind and resorted to if possible.

**Perforation: Its Diagnosis with Reference to Surgical Treatment.**— Since the introduction of operative measures for the relief of this usually fatal complication, increasing stress has been laid on the necessity of early diagnosis for the success of the operation, and attention has been specially directed of recent years to the signs and symptoms which immediately accompany perforation in typhoid fever. It is generally admitted that the combination of signs which constitute "peritonism" in its classical sense, is not met with in this condition until so long a time has elapsed as renders surgical interference hopeless. The peritoneum is unduly tolerant in typhoid fever, and the signs of leaking from the bowel are less marked and much later in their development than in the case of a ruptured gastric ulcer or perforative appendicitis. The sensitiveness of the peritoneum appears to be lessened by the toxæmia of typhoid fever, as exampled by the case of a boy who survived the accident of perforation (as ascertained by immediate operation) for eight days, when an autopsy revealed the fact that his abdomen was full of pus, yet up to the time of his death there was no clear indication of general peritonitis. The difficulties of diagnosing this complication at the time of its occurrence being then so great, what help can be obtained from modern methods and recent observations?

*Pain* is a fairly constant symptom and may be expected to occur in about three-quarters of the cases at the time of the actual perforation, but when this takes place during the height of the fever, with the patient in the true typhoid state, there is little likelihood of any very clear indication of pain; the symptom is most prominent when perforation happens in the early days of convalescence.

*Tenderness and Rigidity* are both commonly met with, but are late in developing, and the value of either sign is discounted by the comparative frequency with which they are observed during the course of the disease apart from perforation; too much attention paid to these signs would undoubtedly lead to hasty and ill-timed operation.

*Distension and Absence of Liver Dullness* lose something of their diagnostic value for similar reasons, they occur apart from perforation, and when this accident has actually taken place, they are frequently too late in their appearance to be of timely assistance.

A steady *fall of temperature* may prove a valuable indication, though the time suggested for its observation by some authorities, namely 4° or 5°, occupying eight or twelve hours, is too long to allow to elapse if operation is to be successfully undertaken.

*The Blood Count*, although at one time held to promise great assistance, has not as a matter of experience proved a sufficiently reliable guide; there is considerable difference of opinion as to how soon leucocytosis may be depended upon to establish itself, and what degree of increase in the leucocytes is to be regarded as consistent with perforation.

There are still left, however, certain indications which may be of the greatest help if present.

When perforation occurs, an inexplicable change in the general *aspect of the patient* is usually to be noted even at the height of the fever; the patient, looking previously, it may be, desperately ill, has suddenly, without warning, without a profuse hæmorrhage or the blanching which accompanies it, appeared moribund. Now this is not consistent with the usual course of typhoid fever, the patient arrives at the point of death gradually, not with the dramatic suddenness of perforation or hæmorrhage. Such an unexpected development in a case of typhoid fever should suggest the occurrence of one or other of these complications.

*The Pulse-rate* affords most valuable and constant information, an increase in the frequency of the pulse being observed in nearly every case of perforation, and that increase a substantial one, varying from 10 to 20 and even 30 beats per minute within a very short time; but this need not be a lasting increase, although in most cases the increased frequency is maintained; occasionally the phase is only transient, the pulse falls again to its former rate, and subsequently increases once more by slower degrees. The sudden and lasting increase is the more usual, but the latter phenomenon of a temporary increase followed by a fall and subsequent slow rise of pulse-rate is not commonly met with apart from perforation. Under either form the importance of the sign is very great, and it is, perhaps, not overstating its value to say that in typhoid fever a sudden increase in the pulse-rate of 10 beats per minute, which cannot be otherwise explained (*e.g.* as part of a progressive cardiac failure, or vomiting, or hæmorrhage) is probably due to perforation.

*The Blood-pressure* is, however, one of the most recently investigated conditions bearing upon the subject, and certainly promises to prove the most reliable indication in perforation. For in the course of an attack of typhoid fever the blood-pressure has been found to be uniformly lowered and to show a tendency to fall steadily during the attack, the average pressure in millimetres of mercury in a long series of cases being 104. Immediately after the event of perforation the blood-pressure has been demonstrated to rise considerably, even reaching 208 mm., while the lowest figure recorded in a series of twenty cases of this complication was 156 mm. This symptom may prove of the utmost value in the early diagnosis of perforation and has the advantage of not requiring elaborate apparatus, while it is already amongst the routine investigations which the physician has added to his clinical methods: the accurate use of the thermometer itself is scarcely less difficult, while the relation of the temperature to the severity of the disease is by no means either constant or instructive.

[1] Deut. Med. Wchnschr., Jan. 11, 1905.

